# Receptor Status Changes, HER2-Low Discordance, and TNBC Transformation in Metastatic Breast Cancer: Prognostic Significance and Impact on Survival Outcomes

**DOI:** 10.3390/jcm15207788

**Published:** 2026-10-09

**Authors:** Ayşe Nuransoy Cengiz, Muhammet Cengiz, Oktay Bozkurt, Gozde Erturk Zararsiz, Aleyna Erakcioglu, Safiye Selcen Vicdanli, Ezgi Yenisekerci Kulak, Ahmet Kursad Disli, Mevlude Inanc, Metin Ozkan

**Affiliations:** 1 Department of Medical Oncology Clinic, Faculty of Medicine, Erciyes University, Yenidogan Mahallesi, Turhan Baytop Street, Talas, Kayseri 38280, Türkiye; 2 Department of Biostatistics, Faculty of Medicine, Erciyes University, Yenidogan Mahallesi, Turhan Baytop Street, Talas, Kayseri 38280, Türkiye; 3 Department of Internal Medicine, Faculty of Medicine, Erciyes University, Yenidogan Mahallesi, Turhan Baytop Street, Talas, Kayseri 38280, Türkiye

**Keywords:** metastatic breast cancer, hormone receptor discordance, HER2 receptor, HER2-low status, triple-negative breast cancer transformation

## Abstract

**Background/Objectives**: Therapeutic options for metastatic breast cancer vary according to hormone receptor (HR) status, human epidermal growth factor receptor 2 (HER2) status, and, importantly, the newly recognized HER2-low status. In triple-negative breast cancer (TNBC), characterized by the absence of HR and HER2 receptors, therapeutic approaches and survival rates differ significantly. Current treatments have improved survival rates in metastatic diseases, resulting in an increased demand for secondary biopsies. The findings from these biopsies affect the range of therapeutic options. **Methods**: The data of 172 patients with metastatic breast cancer were retrospectively analyzed. The initial and subsequent biopsy specimens from these patients were compared. Their effects on survival and prognosis were analyzed statistically. **Results**: Overall, 12.8% of patients transitioned to triple-negative status. Upon analysis of HER2-low status, 13.4% of patients initially classified as HER2-negative transitioned to HER2-low status. The median overall survival was 26 months (95% CI: 19.1–32.8) in the HR-concordant group and 21 months (95% CI: 14.3–27.6) in the HR-discordant group (*p* < 0.03). **Conclusions**: The present study clarifies the rates of HR and HER2 receptor concordance and discordance in metastatic breast cancer. It provides a comprehensive dataset that incorporates all receptors and TNBC transformation. Furthermore, it demonstrates conversion to HER2-low status and examines the implications of TNBC transformation and HR discordance on prognosis and survival.

## 1. Introduction

Recent GLOBOCAN 2022 estimates indicate that breast cancer remains the most commonly diagnosed malignancy among women worldwide, with an annual burden exceeding 2.3 million incident cases and approximately 666,000 deaths [1]. Although outcomes for patients with early-stage breast cancer have improved substantially, recurrence still occurs in nearly 30% of cases, and approximately 5–10% of patients are diagnosed with de novo metastatic disease [2,3].

Breast cancer arises through the accumulation of genetic and epigenetic alterations that result in dysregulated cellular proliferation, survival, differentiation, and invasion. The disease is biologically heterogeneous and can be classified according to histopathological and molecular characteristics [4]. The extent of breast cancer is traditionally assessed using the tumor-node-metastasis (TNM) classification, which evaluates the size and local extension of the primary tumor (T), regional lymph-node involvement (N), and the presence of distant metastases (M). The eighth edition of the American Joint Committee on Cancer (AJCC) staging system further incorporated important biological and pathological characteristics, including tumor grade, oestrogen receptor (ER), progesterone (PR), and human epidermal growth factor receptor 2 (HER2) status, into prognostic stage grouping [5]. Initial evaluation should comprise determining the disease’s stage and analyzing the receptors present on the tumor biopsy specimen. This data is essential for both predictive and treatment-related objectives [6]. Given the heterogeneous nature of metastatic breast cancer, treatment selection is largely based on the tumor’s ER, PR, and HER2 receptor status. Rebiopsy at the time of recurrence has been proposed to identify potential changes in tumor characteristics; however, recommendations differ among current guidelines, and the optimal frequency of biopsy remains uncertain [7,8].

Discordance in these receptors may be detected because only a portion of the tumor may be accessible during the initial biopsy or because the disease may become heterogeneous over time. Data indicate that hormone receptor (HR) and HER2 receptor discordance occurs in 10–40% of cases. This is a wide range, and research on its prognostic significance is ongoing [9,10].

Previous exposure to HR- or HER2-directed therapies may lead to alterations in receptor expression through mechanisms such as receptor downregulation, clonal selection, or the development of acquired resistance [11]. Therefore, reassessment of receptor status at the time of disease progression may provide clinically relevant information for subsequent treatment selection [12]. As therapeutic decisions in metastatic breast cancer are largely guided by the current expression of HR and HER2, changes in receptor status have the potential to influence treatment efficacy, survival outcomes, and overall prognosis [13].

In the present study, we evaluated the rates and importance of receptor discordance between initial and subsequent biopsy specimens obtained during the course of metastatic breast cancer. We further investigated the prognostic significance of receptor conversion and evaluated the factors associated with survival outcomes among patients exhibiting such discordance, aiming to contribute to the existing evidence regarding the clinical implications of changes in receptor status.

## 2. Method

### 2.1. Study Design

**Figure 1:**
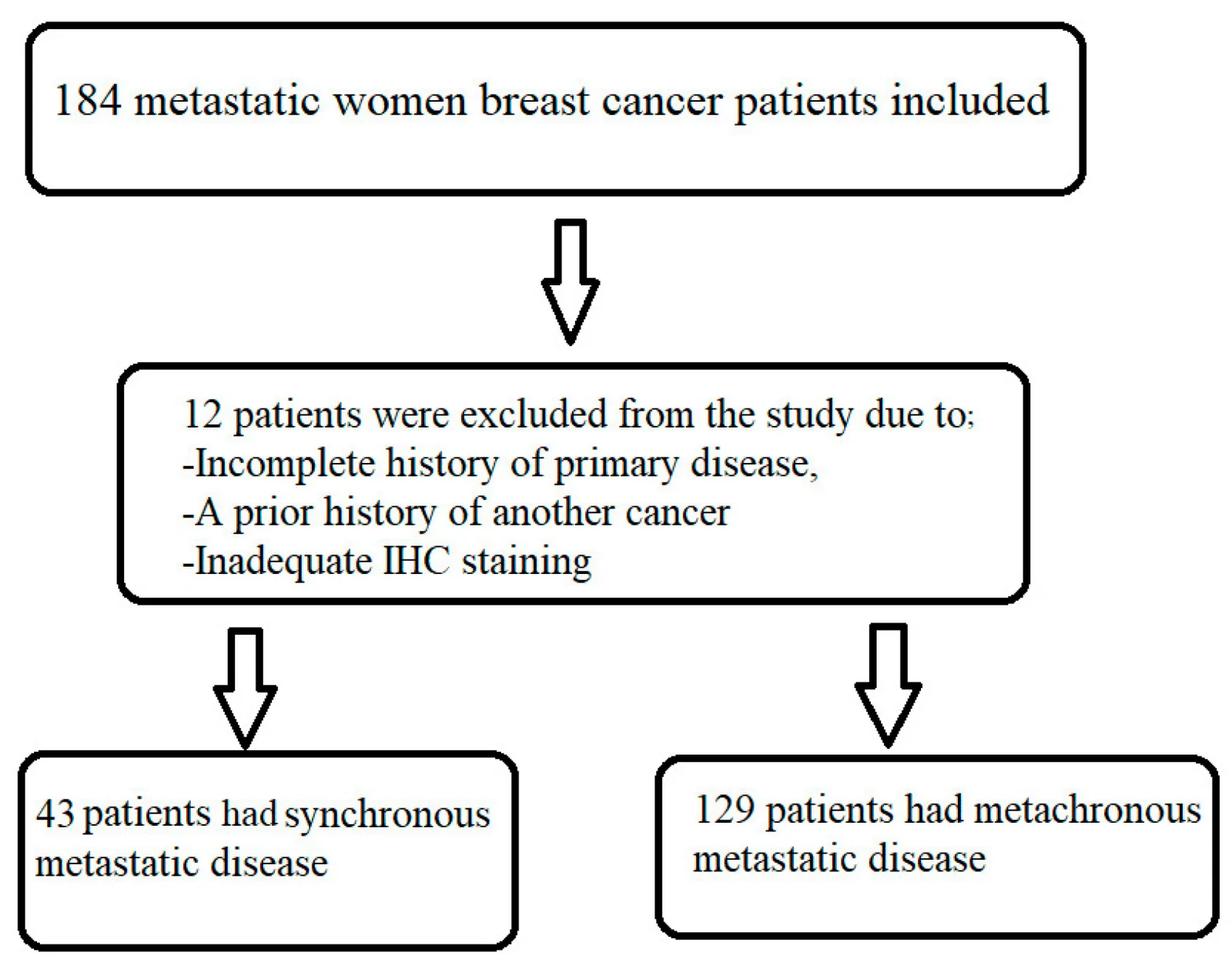
The study population diagram.

Our study included 172 women with metastatic breast cancer who were retrospectively identified. Of these patients, 43 had synchronous metastatic disease and 129 had metachronous metastatic disease. Twelve patients were excluded from the study due to incomplete history of primary disease, a prior history of other cancer and inadequate immunohistochemical (IHC) staining. Data from 172 patients were analyzed (Figure 1). All patients were followed up at the Erciyes University Faculty of Medicine between January 2017 and September 2024.

### 2.2. Data Collection

The inclusion and exclusion criteria for patients were as follows:

Inclusion criteria:

(1) Women aged ≥ 18 years.
(2) The immunohistochemistry (IHC) staining evaluation of ER, PR, and HER2 expression was sufficient.
(3) Diagnosis of invasive carcinoma by fine-needle aspiration or Tru-cut biopsy.
(4) The ability to determine primary and target lesions using positron emission tomography (PET) and computed tomography (CT).
(5) Rebiopsy specimens obtained from target lesions, particularly after disease progression, and the histological evaluation of these tissues.
(6) Availability of clinical and pathological data.

Exclusion criteria

(1) Patients with cancer other than metastatic breast cancer.
(2) Patients with a breast cancer in remission.
(3) Patients with incomplete clinical or pathological information.
(4) Patients who have previously undergone surgery, chemotherapy, or radiotherapy for other cancer diagnosis.
(5) IHC staining evaluation of ER, PR, and HER2 expression were undetermined.

Clinical TNM stage was evaluated. Staging was performed using CT and PET imaging.

ER and PR positivity were defined as nuclear staining up to 1% on IHC examination. HER2 positivity is established by IHC when more than 10% of invasive tumor cells demonstrate strong, circumferential membrane staining (IHC 3+). Alternatively, HER2 amplification can be demonstrated by fluorescence in situ hybridization (FISH), defined by a HER2/CEP17 ratio of ≥2.0 together with an average HER2 copy number of ≥4.0 signals per cell. In addition to its predictive role in treatment selection, HER2 status is incorporated into the AJCC prognostic staging system for breast cancer [14]. HER2 status was defined as IHC 0, IHC 1+, IHC 2+, IHC 3+. In patients with an IHC 2+ result, FISH testing was performed to determine whether the result was positive or negative. Patients with HER2 receptor status of IHC 1+ or IHC 2+, and confirmed as negative by FISH, were classified as HER2-low positive.

Tumor grade was determined using the Nottingham grading system. The Ki-67 proliferation index was assessed using IHC. Ki-67 IHC expression was evaluated using the MIB-1 antibody (Immunotech, Westbrook, ME, USA) as a marker of tumor cell proliferative activity. The Ki-67 labeling index was calculated as the percentage of tumor cells showing positive nuclear staining relative to the total number of tumor cells assessed in each microscopic field. The percentage of tumor cells showing nuclear staining was expressed as a percentage. A cutoff value of 30% was applied. Values below 30% were classified as low proliferation, whereas values of 30% or higher were classified as high proliferation. This cutoff was selected in accordance with the threshold commonly used to define high Ki-67 expression in breast cancer [15].

In particular, in patients with metachronous metastases, previous breast surgical treatments and their types were also examined. The status of breast-conserving surgery or mastectomy and the types of axillary intervention were examined. The pathological TNM stages were assessed. The use of adjuvant radiotherapy and endocrine therapies, including tamoxifen (TMX) and aromatase inhibitors (AIs), was also assessed. Rebiopsies performed during the metastatic period were also evaluated according to receptor status. Changes in receptor status between the rebiopsies and initial biopsies were compared.

### 2.3. Statistical Analysis

Categorical variables summarizing patient demographics, clinical characteristics, and histopathological parameters were presented as frequencies and percentages, *n* (%). Overall survival (OS) was defined as the time interval from the date of the first re-biopsy to death from any cause, with surviving patients censored at the date of their last documented follow-up. Because the time origin coincides with the point at which receptor status at re-biopsy becomes observable, no immortal time is included in the analysis. Median follow-up was estimated using the reverse Kaplan–Meier method, in which the censoring indicator is reversed and the median of the resulting distribution is reported. To investigate the time-dependent effects of potential prognostic factors associated with OS, univariable Cox proportional hazards regression analyses were conducted. The proportional hazards assumption was evaluated for all covariates entered into the Cox models, both graphically by inspection of log-minus-log survival plots and formally by testing time-dependent covariates constructed as the product of each covariate with the natural logarithm of survival time; a non-significant time-dependent term was taken to indicate that the assumption was satisfied. Candidate variables demonstrating a predefined significance threshold of *p* ≤ 0.250 in the univariable analysis were subsequently incorporated into multivariable Cox regression models. During the multivariable phase, basic main-effect models were constructed to explore complex variable dependencies and identify independent predictors of survival. To evaluate time-to-event dynamics, survival curves for OS were estimated utilizing the Kaplan–Meier method, and statistical differences between strata were assessed using the log-rank test.

Specifically, Kaplan–Meier analyses were performed to evaluate the prognostic implications of receptor status dynamics between initial biopsies and subsequent re-biopsies, focusing strictly on overall HR alterations, HER2 status changes, and transitions involving the HER2-low status. All statistical analyses and data management procedures were performed using Statistical Package for the Social Sciences, IBM Corp., Armonk, NY, USA (IBM SPSS Statistics for Windows), Version 25.0, and the R programming language (R Foundation for Statistical Computing, Vienna, Austria; www.r-project.org). A two-sided *p*-value of <0.05 was considered statistically significant.

## 3. Results

A total of 172 women were included in this study. In terms of smoking status, 11% of patients were active smokers, whereas 89% were non-smokers. Among the patients, 57.6% were premenopausal, and 46.3% had a Ki-67 value greater than 30%. Additionally, 43.6% had three or four diseases, and 30.2% had received endocrine therapy. Among patients with metastatic disease, 75% had metachronous metastases and 25% had synchronous metastases. The patient characteristics are summarized in Table 1.

In our patient cohort, 12.8% of patients converted to triple-negative status. Receptor-specific changes were observed as follows: HER2 status converted from negative at the initial biopsy to positive at rebiopsy in 9.3% of patients, whereas it changed from positive to negative in 7.0% of patients. Regarding HER2-low status, 13.4% of patients converted to HER2-low −/+ status, whereas 19.2% of patients converted to HER2-low +/− status. Finally, HR status changed from HR+/− in 17.4% of patients, whereas it changed HR−/+ in 2.9% of patients after rebiopsy (Table 2).

**Figure 2:**
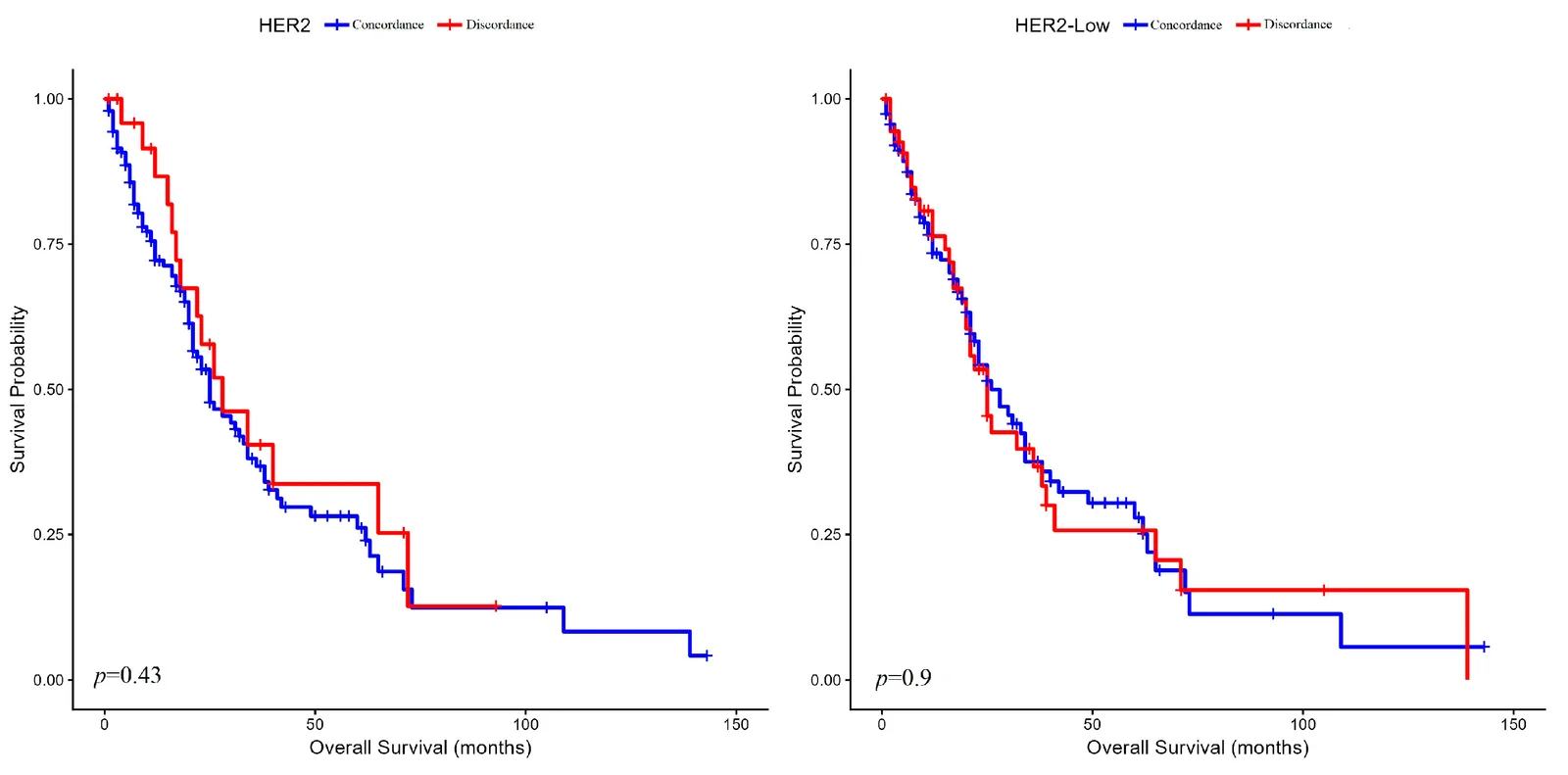
Kaplan–Meier curves for OS according to HER2 and HER2-low receptor discordance and concordance groups.

In our cohort, we observed considerable dynamism in HER2 expression during the metastatic process, including a 9.3% conversion from HER2-negative to HER2-positive status and notable fluctuations within the newly defined HER2-low spectrum (13.4% converting from HER2 negative to HER2-low and 19.2% from HER2-low to HER2 negative). However, despite these high discordance rates, our survival analysis did not identify HER2 or HER2-low status alterations as independent prognostic factors for OS (Figure 2). This lack of prognostic detriment is most likely attributable to the profound efficacy of modern targeted ‘rescue’ therapies. When a tumor converts to a HER2-positive status, the patient rapidly becomes eligible for effective anti-HER2 regimens, which effectively neutralize the biological aggressiveness of the transformed tumor and equalize survival outcomes.

At the time of analysis, 100 of the 172 patients (58.8%) had died and 70 (41.2%) were censored alive at their last documented clinical follow-up. Median follow-up was 39 months (95% CI 32–58). The proportional hazards assumption was satisfied for all covariates included in the Cox models (all time-dependent interaction terms *p* > 0.05). In univariate analysis of OS, significant associations were observed for tumor grade, receipt of adjuvant therapies (including radiotherapy, chemotherapy, and endocrine therapy), HR discordance, and TNBC transformation. Significant variables were tumor grade (*p* = 0.003), receipt of adjuvant chemotherapy (*p* = 0.03), receipt of adjuvant radiotherapy (*p* = 0.02), receipt of adjuvant endocrine therapy (*p* = 0.01), TNBC transformation (*p* = 0.1), and HR discordance (*p* = 0.04) (Table 3). In multivariate analysis, TNBC transformation and HR discordance were significant prognostic factors for OS. Significant variables were as follows: TNBC transformation (HR 1.7, *p* = 0.01) and HR discordance (HR 2.0, *p* = 0.02) (Table 3).

**Figure 3:**
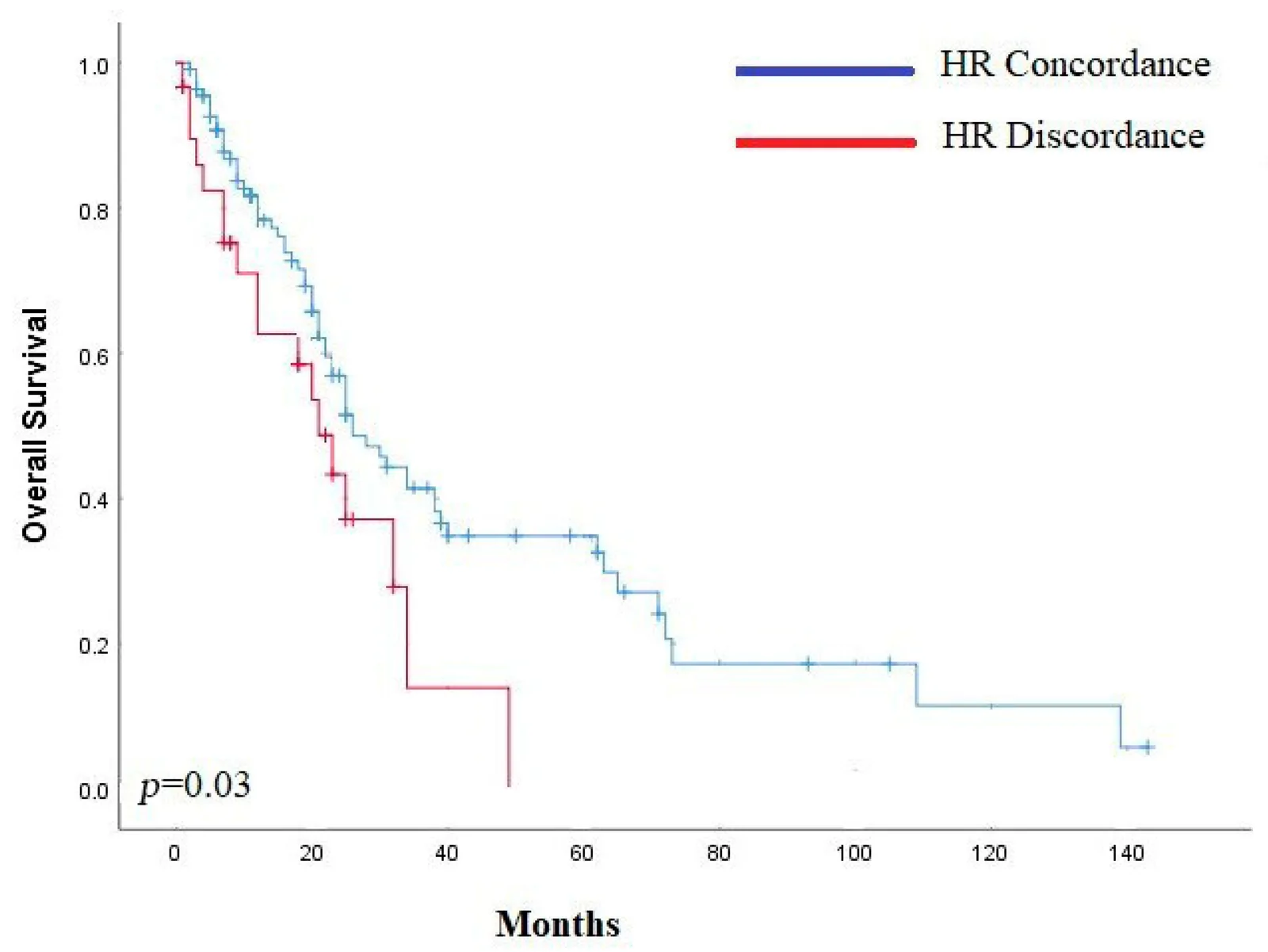
Kaplan–Meier curves for OS according to HR discordance and concordance groups.

Median OS was 26 months (95% CI: 19.1–32.8) in the HR-concordant group and 21 months (95% CI: 14.3–27.6) in the HR-discordant group (*p*  <  0.03) (Figure 3).

## 4. Discussion

Breast cancer is one of the most common cancers among women [16]. In particular, patients receiving treatment during the adjuvant or neoadjuvant phases may develop metastases even after achieving remission [17]. In such cases of disease progression, the treatment may not have failed; rather, alterations in the receptor status targeted by therapy may have occurred. Similarly, in de novo metastatic disease, the initial biopsy may reflect only a specific portion of a heterogeneous tumor [18]. In particular, treatment strategies may be adjusted following rebiopsy and identification of discordance in the setting of disease progression. Although rebiopsy is not explicitly recommended, treatment should be planned according to the receptor status of the metastatic site determined by biopsy [19].

Yang et al. included 270 patients in their retrospective study, all of whom had recurrent breast cancer following treatment. ER negativity was observed in 14% of cases, whereas HER2 negativity was observed in 4%. Importantly, our findings are highly consistent with those of Yang et al., which similarly demonstrated that HR loss and transformation to TNBC are independent predictors of poor OS. In our cohort, 12.8% of patients converted to TNBC, which may be considered a notable proportion. According to the existing data and consistent with the literature, it may be anticipated that patients who transition to TNBC will experience a more aggressive disease course. Therefore, these patients may require closer clinical monitoring.

One of the interesting findings of our study was that 13.4% of patients converted to HER2-low positive status. Contemporary therapies such as transtuzumab deruxtecan are currently used in the treatment of HER2-low individuals [20]. Access to these therapies could extend the lifespan of these individuals. In our study, no variation in survival was observed between patients with and without HER2-low status discordance. This finding may be attributed to the inclusion of patients in our cohort after 2017. Transtuzumab deruxtekan has been utilized since 2022 within the framework of the DESTINY-Breast04 study [21]. Thus, further studies are needed to analyze the survival rates of individuals who have converted to HER2-low status due to receptor discordance.

In a prospective study conducted by Statishkumar et al. [22], loss of receptor expression, rather than its increase, was considered one of the main predictors of poor prognosis. The study included 110 patients, and ER negativity was observed in 26.9% of cases. Discordance was detected most frequently in lung biopsies, followed by liver biopsies. Interestingly, all patients with ER discordance experienced disease progression while receiving adjuvant treatment. In our study, 17.4% of patients who were initially HR-positive converted to HR-negative status. HR discordance may significantly affect survival outcomes and may serve as an independent prognostic factor. Therefore, subsequent biopsies, particularly in the metastatic setting, may provide information for clinical decision-making and potentially alter the treatment strategy for 17.4% of patients.

A meta-analysis including 15 studies and 1373 patients with brain metastases reported that HR negativity in brain metastases was observed in 40% of cases [23]. This rate was considerably higher than that observed in our cohort. However, this meta-analysis only compared brain biopsies and did not address discordance in subsequent biopsies. In contrast, in our study, the second biopsy was also performed on other solid organs. As the disease becomes more aggressive, receptor loss and the upregulation of growth factors may occur. This process, in turn, reduces survival [24].

Contrary to previous studies, the Ki-67 index was not identified as a prognostic factor in our study. In the univariate analysis, a higher grade was associated with poorer outcomes [25]. Thus, a higher grade may offer a more accurate representation than Ki-67 index when evaluating patients.

Nonetheless, despite the retrospective design of our study, it provides a considerable follow-up duration of seven years and relies upon real-world data. This study considerably contributes to the literature by illustrating the progression of metastatic breast cancer and its receptor status.

In conclusion, our study clarifies the frequencies of HR and HER2 receptor concordance and discordance in metastatic breast cancer. It includes an extensive dataset encompassing all receptors and TNBC transformation. Moreover, it demonstrates conversion to HER2-low status, suggesting that a considerable proportion of patients may benefit from current therapies such as transtuzumab deruxtecan. Additionally, it scrutinizes the effects of TNBC transformation and HR discordance on prognosis and survival. All these up-to-date findings aim to contribute to the literature and provide practical information for clinicians in the management of patients.

## Figures and Tables

**Table 1:** Characteristics of participants according to HR Groups.

Variables	*HR* Groups		Total (*n* = 172)	*p*-Value
	Concordance *n* = 137 (%)	Discordance *n* = 35 (%)		
Smoking status				0.70
No	123 (89.8)	30 (85.7)	153 (89)	
Yes	14 (10.2)	5 (14.3)	19 (11)	
Menopausal status				0.74
Premenopausal	78 (56.9)	21 (60)	99 (57.6)	
Postmenopausal	59 (43.1)	14 (40)	73 (42.4)	
Histology				0.97
Invasive ductal carcinoma	125 (91.2)	32 (91.4)	157 (91.3)	
Invasive lobular carcinoma	12 (8.8)	3 (8.6)	15 (8.7)	
Ki 67 status				0.81
<30	74 (54.2)	21 (60)	95 (53.7)	
≥30	63 (45.8)	14 (40)	77 (46.3)	
Grade				0.86
1–2	78 (56.9)	19 (54.3)	97 (56.4)	
3–4	59 (43.1)	16 (45.7)	75 (43.6)	
Endocrine Therapy				0.14
No	97 (70.8)	23 (65.7)	120 (69.8)	
Yes	40 (29.2)	12 (34.3)	52 (30.2)	
Metastatic Status				0.32
De novo	32 (23.4)	11 (31.4)	43 (25)	
Metachronous	105 (76.6)	24 (68.6)	129 (75)	
Metastatic sites				0.55
Visceral	88 (64.2)	24 (68.6)	112 (65.1)	
Lymph node	19 (13.9)	2 (5.7)	21 (12.2)	
Bone	16 (11.7)	5 (14.3)	21 (12.2)	
Breast	14 (10.2)	4 (11.4)	18 (10.5)	
HER2 status at first biopsy				0.52
Negative	105 (76.6)	25 (71.4)	130 (75.6)	
Positive	32 (23.4)	10 (28.6)	42 (24.4)	
HR status at first biopsy				0.69
Negative	26 (19)	5 (14.3)	31 (18)	
Positive	111 (81)	30 (85.7)	141 (82)	

Values are presented as *n* (%). HR: Hormone receptors.

**Table 2:** Characteristics of participants according to receptor status changes.

Variables	Frequency (*n*)	Percent (%)
Hormone receptor status change		
Negative to negative	26	15.1
Negative to positive	5	2.9
Positive to negative	30	17.4
Positive to positive	111	64.5
HER2 status change		
Negative to negative	114	66.3
Negative to positive	16	9.3
Positive to negative	12	7.0
Positive to positive	30	17.4
HER2-low status change		
Negative to negative	106	61.6
Negative to positive	23	13.4
Positive to negative	33	19.2
Positive to positive	10	5.8
Transformation to TNBC		
No	150	87.2
Yes	22	12.8

TNBC: Triple-negative breast cancer. Values are expressed as *n* (%).

**Table 3:** Univariate and multiple Cox regression analyses of variables associated with OS.

Variables	Univariate		Multivariate	
	HR (95%CI)	*p*-Value	HR (95%CI)	*p*-Value
First biopsy Ki-67 index (%)				
<30	1.00	-		
≥30	1.4 (0.8–2.5)	0.3		
HER2-low-positive status				
No	1.00	-		
Yes	0.7 (0.4–1.2)	0.2		
Grade				
1–2	1.00	-		
3–4	3.3 (1.1–10.0)	**0.003**		
Adjuvant chemotherapy				
No	1.00	-		
Yes	0.5 (0.2–0.9)	**0.03**		
Adjuvant endocrine therapy				
No	1.00	-		
Yes	0.2 (0.1–07)	**0.01**		
Adjuvant radiotherapy				
No	1.00	-		
Yes	0.5 (0.3–0.9)	**0.02**		
Brain metastasis				
Yes	1.00	-		
No	1.0 (0.5–1.1)	0.9		
Reason for rebiopsy				
Synchronous progression	1.00	-		
Metachronous progression	0.5 (0.3–1.0)	0.06		
Transformation to TNBC				
No	1.00	-	1.00	-
Yes	1.7 (0.8–3.3)	**0.1**	2.0 (1.3–3.3)	**0.01**
Hormone receptor status change				
No	1.00	-	1.00	-
Yes	2.0 (1.1–3.3)	**0.04**	2.5 (1.1–5.0)	**0.02**

CI: Confidence interval; HR: Hazard ratio; OS: Overall Survival, TNBC: Triple-negative breast cancer; HER2: Human epidermal growth factor receptor 2. Statistically significant *p* values are shown in bold.

## Data Availability

The datasets generated and/or analyzed during the current study are available from the corresponding author upon reasonable request.

## Abbreviations

HR	Hormone receptor
HER2	Human Epidermal Growth Factor Receptor 2
ER	Oestrogen receptor
PR	Progesterone receptor
TNBC	triple-negative breast cancer
TNM	Tumor-node-metastasis
T	Primary tumor
N	Lymph-node involvement
M	Distant metastases
IHC	Immunohistochemical
TMX	Tamoxifen
AIs	Aromatase inhibitors
OS	Overall survival
PET	Positron emission tomography
CT	Computed tomography
FISH	Fluorescence In Situ Hybridization
